# Comparative conventional- and quantum dot-labeling strategies for LPS binding site detection in *Arabidopsis thaliana* mesophyll protoplasts

**DOI:** 10.3389/fpls.2015.00335

**Published:** 2015-05-12

**Authors:** Londiwe S. Mgcina, Ian A. Dubery, Lizelle A. Piater

**Affiliations:** Department of Biochemistry, University of Johannesburg, JohannesburgSouth Africa

**Keywords:** defense, flow cytometry, innate immunity, lipopolysaccharides (LPSs), LPS-binding sites, LPS-labeling, microbe-associated molecular pattern (MAMP), protoplasts

## Abstract

Lipopolysaccharide (LPS) from Gram-negative bacteria is recognized as a microbe-associated molecular pattern (MAMP) and not only induces an innate immune response in plants, but also stimulates the development of characteristic defense responses. However, identification and characterization of a cell surface LPS-receptor/binding site, as described in mammals, remains elusive in plants. As an amphiphilic, macromolecular lipoglycan, intact LPS potentially contains three MAMP-active regions, represented by the O-polysaccharide chain, the core and the lipid A. Binding site studies with intact labeled LPS were conducted in *Arabidopsis thaliana* protoplasts and quantified using flow cytometry fluorescence changes. Quantum dots (Qdots), which allow non-covalent, hydrophobic labeling were used as a novel strategy in this study and compared to covalent, hydrophilic labeling with Alexa 488. Affinity for LPS-binding sites was clearly demonstrated by concentration-, temperature-, and time-dependent increases in protoplast fluorescence following treatment with the labeled LPS. Moreover, this induced fluorescence increase was convincingly reduced following pre-treatment with excess unlabeled LPS, thereby indicating reversibility of LPS binding. Inhibition of the binding process is also reported using endo- and exocytosis inhibitors. Here, we present evidence for the anticipated presence of LPS-specific binding sites in *Arabidopsis* protoplasts, and furthermore propose Qdots as a more sensitive LPS-labeling strategy in comparison to the conventional Alexa 488 hydrazide label for binding studies.

## Introduction

Lipopolysaccharides (LPSs) are complex lipoglycans found in the outer membrane of Gram-negative bacteria and is generally composed of three regions namely the fatty acid lipid A disaccharide, a core region of short oligosaccharide chains and an O-antigen region of polysaccharides (Madala et al., 2011, 2012). This composition, however, varies among different bacterial species and strains. The O-antigen moiety may be shortened or absent to result in a more hydrophobic molecule (rough LPS), while smooth LPS has a more amphiphilic nature due to a repetitive glycan polymer.

Lipopolysaccharide elicits toxic- and inflammatory responses in mammals due to lipid A, lipid A precursors and the covalently-linked core region which possess immunogenic properties (Silipo et al., 2005). This potent biological response to LPS is triggered *via* receptor-mediated recognition. Here, LPS binds to a LPS binding protein (LBP) to form a LPS–LBP complex which is translocated to myeloid differentiation 2 (MD2) with the presence/absence of its co-receptor, a glycosylphosphatidylinositol (GPI)-linked protein, CD14 (Triantafilou et al., 2001; Sasaki and White, 2008). The interaction occurs on the host membrane, and triggers a signaling cascade activated by the interaction with Toll-like receptor 4 (TLR4; Triantafilou et al., 2001; Scott et al., 2009). The latter, a leucine-rich repeat (LRR) protein, acts as a pattern-recognition receptor (PRR) for perception of LPS as a microbe-associated molecular pattern (MAMP) molecule, and initiates transduction of ligand-specific perception, subsequent signaling cascades and the activation of an immune response (Nürnberger et al., 2004; Albright et al., 2009; Betanzos et al., 2009).

Lipopolysaccharide is recognized as “non-self” (Van Loon et al., 1998; Sanabria et al., 2008; Dubery et al., 2012) and has, to date, been shown to act as a MAMP which induces defense responses in plants (Coventry and Dubery, 2001; Zeidler et al., 2004; Erbs and Newman, 2012). These include activation of an oxidative burst characterized by the release of reactive oxygen species (ROS), a nitric oxide (NO) burst, an influx of Ca^2+^ ions, extracellular medium alkalinization and reversible protein phosphorylation *via* mitogen-activated protein kinases (MAPKs; Gerber et al., 2004, 2006; Piater et al., 2004; Zeidler et al., 2004), resulting in activation of defense genes (Zeidler et al., 2004; Madala et al., 2011, 2012). Increased expression of receptor-like kinases (RLKs), such as the rapid biphasic induced-response of *Nt*-S*d*-*RLK* (Sanabria et al., 2012) has also been reported. Systemic acquired resistance (SAR) is furthermore known to be triggered by LPS elicitation through in the expression of *PR* genes in upper leaves upon lower leaf treatment (Coventry and Dubery, 2001; Zeidler et al., 2004; Mishina and Zeier, 2007). Such defense components lead to heightened plant sensitivity to subsequent stimuli and microbe sensing, thus termed a primed state (Newman et al., 2007; Sanabria et al., 2008; Madala et al., 2012).

The mechanism whereby plants perceive LPS is, however, not known, and all three structural components have similarly been shown to induce defense responses (Erbs and Newman, 2012; Madala et al., 2012). This thus questions the sole role of lipid A as seen in mammalian perception and introduces a focus on other LPS subcomponents. Furthermore, it would be incorrect to assume that LPS passively diffuses through the plant cell plasma membrane due to its large size and amphipathic nature (Gross et al., 2005), and thus it most likely interacts with a surface-localized receptor or receptor complex as the primary binding site. Here, two modes of perception are possible, namely direct recognition and binding of the LPS by a PRR(s), or indirectly as a result of ligand-induced conformational changes, dimerization, and/or recruitment of a co-receptor. The latter then results in auto- and trans-phosphorylation of the receptor as well as other proteins (Altenbach and Robatzek, 2007; Chinchilla et al., 2007; Zipfel, 2009).

A number of LPS-labeling strategies have been employed. In conjunction with flow cytometry, 4,4-difluoro-4-bora-3a,4a-diaza-*s*-indacene [or boron-dipyrromethene (BODIPY)], as well as Alexa 488 hydrazide, biotin, and fluorescein isothiocyanate (FITC), have successfully been used in mammalian studies (Odeyale and Kang, 1988; Triantafilou et al., 2000; Betanzos et al., 2009). In comparison, FITC-labeling was investigated in plant cells (*Nicotiana tabacum*) in endocytosis investigations of LPS from *Xanthomonas campestris* pv. *campestris* (Gross et al., 2005), while Alexa 488-labeling was employed in mobility studies of LPS from *Salmonella minnesota* in *Arabidopsis*. These approaches modify chemical groups (particularly the hydroxyl groups of the O-antigen) upon conjugation. Pathogens that lack an O-antigen, such as *X. campestris* pv. *campestris*, have a rough LPS component and since Alexa 488 hydrazide readily interacts with hydrophilic components, labeling is impossible due to smooth LPS being preferred (Triantafilou et al., 2000). As such, an alternative labeling strategy is required like the use of quantum dots (Qdots). Betanzos et al. (2009) described the labeling of both lipid A and lipoglycan (LPSs from *Escherichia coli* and *Pseudomonas aeruginosa*) using Qdot-conjugation through hydrophobic interactions.

Since most LPS labels, including Qdots, are fluorophores, flow cytometry can be used for quantification of cell–ligand binding interactions through the analysis of optical properties such as fluorescence and light scatter at a specific emission wavelength. This technique, however, requires single cells, and thus the production of protoplasts from higher plant tissues (Galbraith, 1994; Doležel et al., 2007; Yoo et al., 2007).

In this study we compare LPS-labeling with Alexa 488 to Qdots as covalent and non-covalent hydrophobic conjugating strategies respectively, for binding studies in *Arabidopsis thaliana* mesophyll protoplasts. Besides comparison between these, more notably we report for the first time, both conjugates showed affinity for LPS-specific binding sites. Inhibitor studies furthermore suggest that the binding site-ligand interaction may be subject to binding site recycling *via* endo- and exocytosis processes similar to that reported for MAMP receptors (Zipfel, 2014).

## Materials and Methods

### Protoplast Isolation and Determination of Viability

Seeds of *A. thaliana* (Columbia ecotype) were planted in soil and allowed to germinate in an environment-controlled plant growth room at 24°C with a 8 h light/16 h dark photoperiod to generate mature plants. For protoplast isolation, 2 g *A. thaliana* leaves were cut into 1–2 mm strips and the protocol according to Yoo et al. (2007) was followed using enzymatic digestion with a solution containing cellulase “Onosuka”R10, macerozyme R10 (Yakult Pharmaceutical Industry, Tokyo, Japan) and pectinase (Sigma, St. Louis, MO, USA). Micrographs were taken to show the disintegration of clumped protoplasts at regular 30 min intervals for 2 h. The obtained protoplasts were resuspended in 5 ml MMg buffer (0.4 M mannitol; 15 mM MgCl_2_; 4 mM MES, pH 5.7) and the concentration determined with the use of a hemocytometer slide (Boeco, Germany) so as to obtain a working concentration of 2 × 10^5^/ml at room temperature (RT). The integrity of protoplasts was monitored over a 32 h time period using the fluorescein diacetate (FDA) stain according to Cheng and Bélanger (2000). A stock solution of 5 mg/ml FDA was prepared in acetone, and a 2 × 10^5^/ml protoplast suspension incubated with 50 μg/ml stain at RT for 10 min. Protoplast micrographs were taken on a Carl Zeiss fluorescent microscope under 430–490 nm fluorescence filters, and live (yellow) and dead (red) protoplasts viewed and counted (Fernandez-DaSilva and Yuffa, 2006).

### Lipopolysaccharide-Labeling and Characterization

Lipopolysaccharide from *E. coli* Serotype O55:B5 (LPS*_E. coli_*; Sigma, St. Louis, MO, USA), Alexa Fluor^®^ 488 conjugate (Alexa-LPS*_E. coli_*; Eugene, Oregon, USA), and Qdot 605 ITK in decane (Invitrogen) were used for the investigations. The commercial Alexa-LPS*_E. coli_* was used without further modification. Conjugation of LPS*_E. coli_* to quantum dots (Qdots) was performed according to Morales-Betanzos et al. (2011). This method is reported to have broad applicability in studies that require LPS bio-distribution visualization and in the identification of an LPS-binding site in mammals.

### Characterization of Quantum Dot-LPS Conjugates by Transmission Electron Microscopy (TEM) Analysis and Absorbance Spectral Scans

Several protocols can be employed to characterize Qdot-LPS conjugates, with TEM chosen as the most reliable technique due to visualization of conjugates and the ability to determine the diameter of complexes formed. This analysis was employed in the characterization of Qdot-LPS*_E. coli_* conjugates, LPS*_E. coli_* in MMg buffer, and Qdots in chloroform (both latter samples serving as negative controls) since the hydrophobic nature of the organic solvent prevents the formation of micelles (Betanzos et al., 2009). Five μl of each sample was placed on a carbon formvar mesh grid, and air dried. Negative staining was employed with the use of uranyl acetate producing a high electron density and image contrast. High resolution images were obtained using an Olympus Veletta camera operating at an acceleration voltage of 200 kV (Department of Microscopy, University of Pretoria, South Africa). Absorbance spectral scans of all samples was also determined using a fluoroscan instrument with excitation set at 485/20 and emission at 590/35 nm.

### Quantification of Quantum Dot-LPS Conjugates by 2-Keto-3-Deoxyoctonate (KDO) Determination

Quantum dot-conjugates can be quantified by molarity and mass concentration of LPS, however, due to size heterogeneity of the lipoglycan not being well defined (personal communication with D. Maysinger, Lalancette-Herbert et al., 2010), it is not possible to determine the exact molar concentration of LPS conjugates. As an alternative, indirect determination of the LPS concentration through the quantification of KDO in the conjugate (Barr et al., 2010) was used as a quantitative marker. This also served to comparatively assess the concentration of LPS bound to Qdots in relation to the Alexa-LPS*_E. coli_* binding studies. The KDO assay was performed according to Karkhanis et al. (1978) and the content calculated (Coventry and Dubery, 2001).

### Optimization of Protoplast Treatment and Flow Cytometry Measurements

The variation of several parameters was investigated in order to determine the optimal binding concentration, temperature and incubation time of Alexa-LPS*_E. coli_* and Qdot-LPS*_E. coli_*, respectively, in *Arabidopsis* mesophyll protoplasts. According to Venis (1985) the ideal concentration range in ligand-receptor studies is that which will saturate between 10 and 90% of the receptor. As such, an Alexa-LPS*_E. coli_* concentration range was investigated between 0.18 and 1.0 μg/ml, while that for Qdot-LPS*_E. coli_* was examined between 0.11 and 0.46 μg/ml. These ranges were selected firstly to allow comparison between the alternatively labeled lipoglycan-conjugates. A second consideration was that the critical aggregation concentrations (CACs) of various LPSs are reported to be between 11 and 22 μg/ml, and this would lead to clumping of labeled protoplasts and hence impede flow cytometry measurements (Aurell and Wistrom, 1998). Lastly, at FITC-labeled-LPS*_X.c.c_* concentrations as low as 5 μg/ml uniform binding on the cell surface was already observed 10 min post-treatment followed by significant internalization after 30 min in *N. tabacum* cell suspensions (Gross et al., 2005). Thus to follow binding over time, a 10-fold lower concentration was selected. The optimal treatment incubation temperature was similarly investigated at 4°C, 20–22°C (RT), 37°C and 65°C respectively, whereas the optimal incubation time ranged from 10 to 120 min. In all cases, protoplasts (2 × 10^5^/ml) were placed on a rotary shaker at 20 rpm, with protoplasts in MMg solution serving as an untreated negative control sample. The mean fluorescence intensity of all the samples was measured on a BD FACS Aria flow cytometer (Becton, Dickenson and Company, Germany) using a 100 μm diameter nozzle to accommodate protoplasts with diameters ranging from 30 to 80 μm, equipped with a blue, red and violet laser. The instrument was excited at 488 nm by the blue laser and filtered on the FITC channel at bandpass (BP) 530/30 (thus light transmitted between 500 and 560 nm) for Alexa-LPS*_E. coli_* samples, while for Qdot-LPS*_E. coli_* the Texas Red channel at BP 610/20 was used (thus light transmitted between 590 and 630 nm) to determine the mean fluorescence of each sample. Following optimization, a constant number of events was counted for each analysis and samples are reported relative to the control value which is set at 100% (the mean fluorescence values of individual controls ranged from 3500 to 28000).

### Binding Site Studies

Binding site studies were performed to investigate (i) concentration-, temperature-, and incubation-time dependence (*in vitro* controlled parameters), (ii) dose-response relationship and (iii) reversibility of the administered ligand. Following optimization of the *in vitro* parameters with the respective labeled LPS-conjugates, the dose-response relationship was determined by isolating protoplasts and dilution to six concentrations namely 1.2 × 10^5^, 2 × 10^5^, 4 × 10^5^, 8 × 10^5^, 1.2 × 10^6^, and 1.6 × 10^6^ protoplasts/ml. Here, the option to vary the protoplast- and not LPS-concentration is again ascribed to the CMC and internalization concerns addressed in the optimal concentration studies. The protoplasts were then treated with the determined concentration of labeled LPS as specified in the figure legends. Reversibility of binding was determined by pre-treatment of 2 × 10^5^ protoplasts/ml with unlabeled LPS (up to 100X excess) for 30 min at RT on a rotary shaker set at 20 rpm, followed by elicitation with labeled LPS-conjugates (as determined), with negative (unlabeled LPS) and positive (respective labeled LPS*_E. coli_*) controls for comparison. All resulting data were based on the measured relative fluorescence determined as described.

### Exo- and Endocytosis Inhibition of Labeled LPS*_E. coli_* Binding Site Studies

Based on previous work (Gross et al., 2005; Robatzek et al., 2006; Beck et al., 2012b), it was deemed necessary to determine whether LPS could be endocytosed into the plant cell (protoplast) to trigger the induction of a defense response, or whether the defense response occurs due to interactions with binding site-/receptor-like proteins on the membrane. Fifty μg/ml Wortmannin in dimethyl sulfoxide (DMSO) and Brefeldin A (BFA) in ethanol respectively, were thus used to investigate endo- and exocytosis inhibition. Co-treatment of protoplasts (2.5 × 10^5^ protoplasts/ml in MMg buffer) involved simultaneous treatment with either Wortmannin or BFA and labeled LPS*_E. coli_*-conjugate, followed by incubation at RT for 120 min. Sole Wortmannin, BFA and unlabeled LPS*_E. coli_* treatments were included as controls.

## Results and Discussion

### Integrity of Protoplasts following Isolation

**FIGURE 1:**
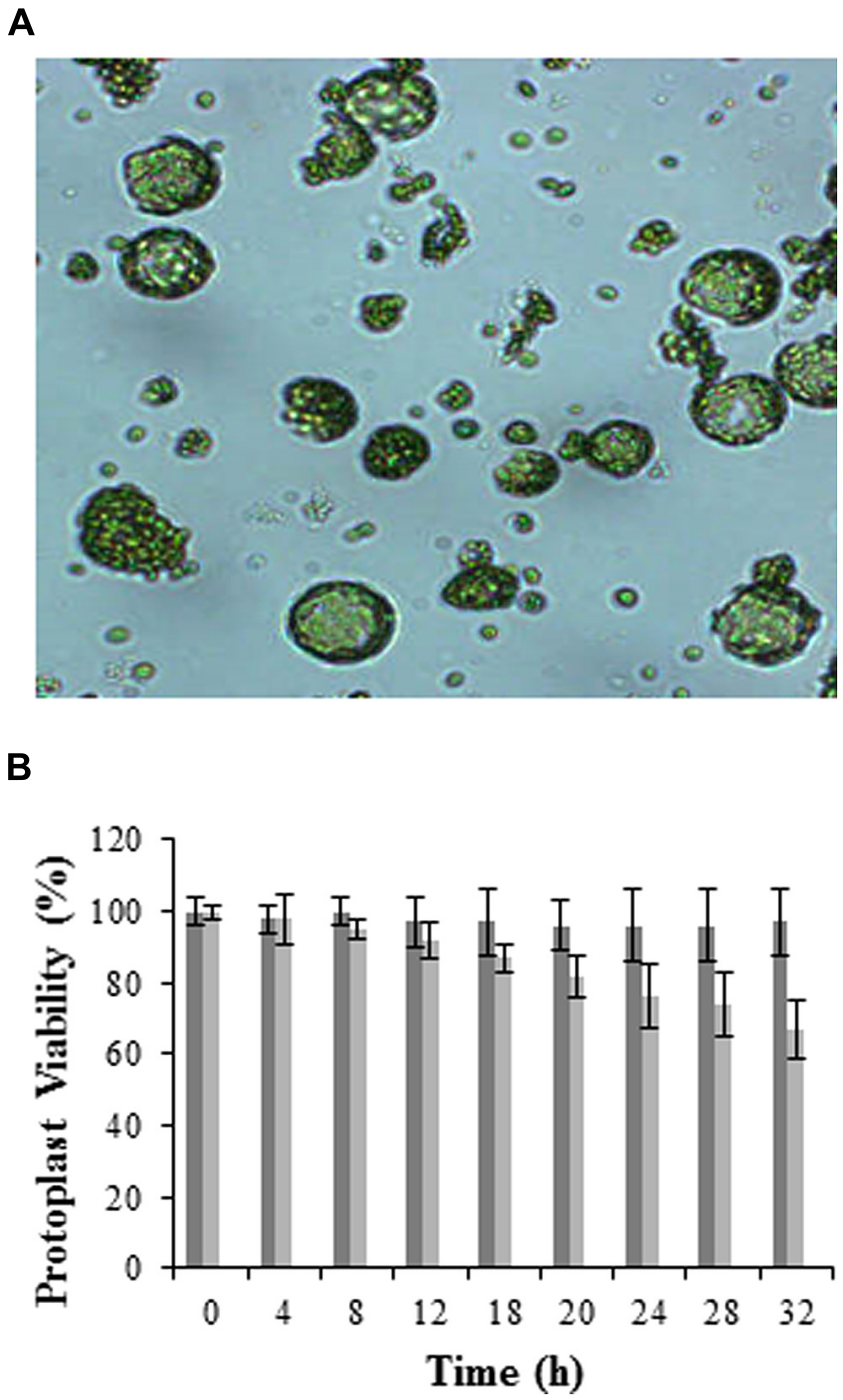
**Visualization and viability of mesophyll protoplasts isolated from *Arabidopsis* leaves. (A)** Micrograph showing protoplasts at 100X magnification (micrographs were taken to follow the digestion process prior to washing and hence debris is still visible), and **(B)** histogram illustrating viability over time using the FDA stain. Dark gray bars represent total protoplasts, while light gray bars represent viable protoplasts, with error bars indicating the standard deviation of three independent biological repeats relative the control set at 100%.

When optimally isolated, protoplasts retain cell integrity as well as original biochemical and physical properties (Galbraith, 1994; He et al., 2006), thus making these structures attractive for use in plant studies. Although several groups have attempted other methods for isolating protoplasts such as the use of a sandwich tape method (Wu et al., 2009), the enzymatic digestion protocol is still preferred and was used in this report. Results obtained show a homogenous population of mesophyll protoplasts isolated from *Arabidopsis* having an approximate diameter between 30 and 60 μm (**Figure 1A**). Although mesophyll protoplasts autofluorescence with emission between 400 and 600 nm due to the chlorophyll content, these were preferred over undifferentiated cultured cells since the study pertained to the perception of LPS in aerial plant tissue, i.e., specifically in plant leaves (Zeidler et al., 2010; Beets et al., 2012).

Cell viability was determined directly through the presence of cytoplasmic esterases that hydrolyze non-fluorescent FDA (Horvath, 2009) to free fluorescent fluorescein molecules within the cell (Fernandez-DaSilva and Yuffa, 2006). Results in **Figure 1B** are represented as 100% viability for the control (0 h) which is composed of freshly isolated protoplasts at high yields without cell breakage or osmotic shrinkage, and a 20% relative loss in viability at 24 h when protoplasts are incubated at RT. Longer time periods (>24 h) resulted in a significant loss in protoplast viability as seen in other investigations (Liqing et al., 2005; Zhang and Wong, 2011). Protoplasts were thus prepared immediately prior to experimentation.

### Quantum Dot (Qdot)-labeling of LPS, and Characterization and Quantification of the Conjugate

Alexa 488 hydrazide-labeling of LPS involves modification of the hydroxyl groups, particularly the O-antigen, as previously used in binding and mobility studies in mammalian cells and *Arabidopsis* leaves respectively. Alexa 488 is thus able to conjugate to hydrophilic LPS containing an O-antigen (‘smooth’ LPS), but not to hydrophobic ‘rough’ LPS [lipid A moiety attached to an inner/outer core or lipooligosaccharide (LOS); Triantafilou et al., 2000]. As such, an alternative strategy employing Qdots was also included. Qdots may be synthesized as highly hydrophobic structures and thus used to target the hydrophobic lipid A, common to both LPS and LOS. Furthermore, these hydrophobic, nanometer-sized molecules can be made water-soluble and biocompatible (Chan and Nie, 1998) as illustrated by the Qdot-LPS*_E. coli_* conjugate TEM micrographs shown in Supplementary Figures S1F–S1I in comparison to Qdots in a chloroform organic control in Supplementary Figures S1A–S1C. Kim et al. (2008) reported that Qdots exist as scattered molecules in organic solution and as ordered structures when conjugated to ligands because of their resulting hydrophilicity that supports the formation of water-soluble structures. We were able to report the same for Qdots in chloroform and when conjugated to amphipathic LPS*_E. coli_* in comparison to LPS in an aqueous solution. Also, such micelle formations with an approximate diameter of 100 nm correlate well with those reported by Betanzos et al. (2009) and Lalancette-Herbert et al. (2010).

Absorbance spectral scans were further used to characterize the Qdot-LPS*_E. coli_* conjugate. The Qdot^®^ 605 ITK^TM^ nanoparticles show a peak emission wavelength of 605 nm, and at this wavelength both Qdots and Qdots-LPS*_E. coli_* samples illustrated a peak (Supplementary Figure S2), thus demonstrating that the conjugate maintained the absorptive properties of Qdots, and so the use thereof was further supported.

Various methods exist for quantification of the molarity of Qdot-conjugates if synthesized in-house (Yu et al., 2003; Lalancette-Herbert et al., 2010). However, the current study utilized commercial Qdots without any known characteristics except for the hydrophobic nature. Also, the molar mass of commercial LPS is not known due to size heterogeneity of preparations For these reasons, the concentration of unlabeled LPS was indirectly determined by the KDO content as 5.25 μg KDO mg^-1^ LPS*_E_.coli* and used to extrapolate the concentration of the prepared Qdot-LPS*_E. coli_* conjugate.

### Optimization of Protoplast Treatment and Flow Cytometry Measurements

An investigation into the optimal conditions for conjugate-LPS*_E. coli_* binding studies was essential, and required various parameters including conjugate concentration, treatment temperature, and incubation time to be explored. The optimal labeled LPS*_E. coli_* concentration was determined over a range by studying the fluorescence response exhibited for each conjugate at a protoplast concentration of 2.5 × 10^5^/ml.

**FIGURE 2:**
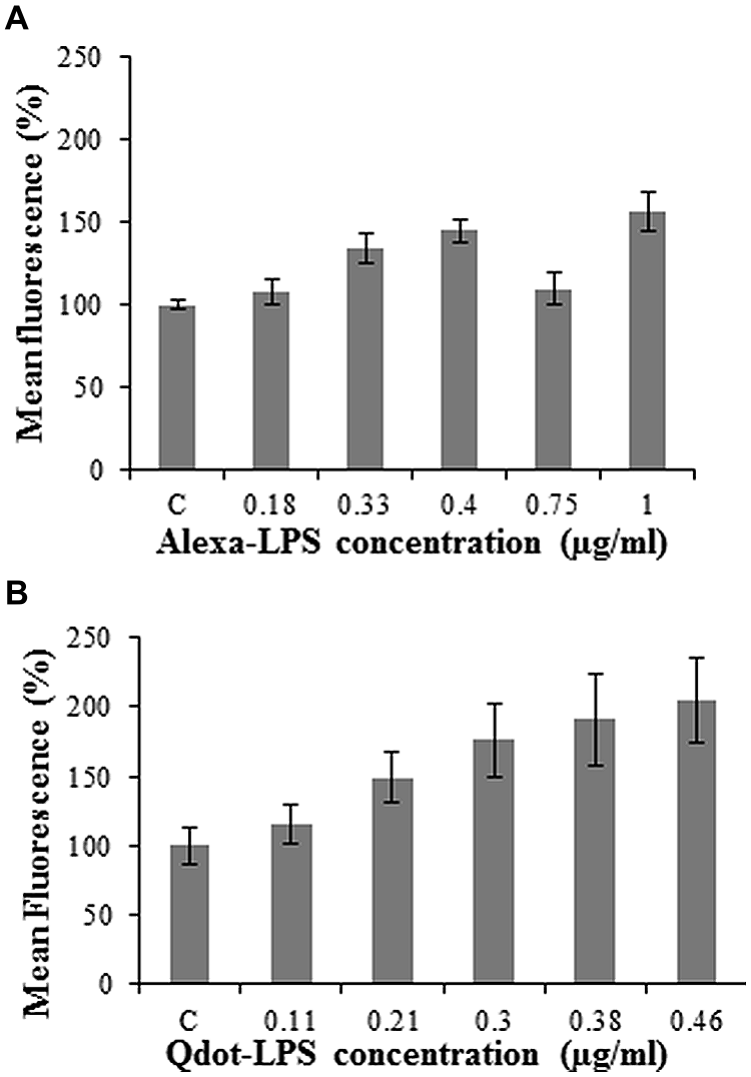
**Optimal concentration determination of LPS-conjugates in *Arabidopsis* protoplast binding studies incubated for 120 min with **(A)** Alexa-LPS*_E. coli_* and **(B)** Qdot-LPS*_E. coli_***. The control (C) represents 2 μg/ml unlabeled LPS*_E. coli_-*treated *Arabidopsis* mesophyll protoplasts at a concentration of 2.5 × 10^5^/ml set at 100%. Each data point represents the mean of three independent experiments and error bars the standard deviation thereof.

Using flow cytometry, events were counted for each conjugate concentration treatment and the fluorescence within the protoplast population gate was recorded. The unlabeled LPS*_E. coli_-*treated protoplasts served as the control set at 100% so as to easily differentiate between samples. The lowest optimal Alexa-LPS*_E. coli_* concentration (**Figure 2A**) from different plant protoplast samples was determined to be 0.4 μg/ml. It is unclear why a concentration of 0.75 μg/ml would produce a lower signal in this case, but was consistent over a number of experimental repeats. From **Figure 2B**, a concentration of 0.46 μg/ml Qdot-LPS*_E. coli_* was shown to be optimal and thus selected for subsequent treatments as well as for comparison with the Alexa-LPS*_E. coli_* (0.4 μg/ml) binding studies. This concentration, although slightly higher than that of the Alexa-LPS*_E. coli_*, was selected since quantification of the Qdot-conjugate was extrapolated from an indirect KDO assay, and is thus not an absolute quantification. Also, both LPS conjugates were individually measured by flow cytometry, however, no fluorescence was detected (data not shown). The concentration study results cannot be compared to literature since similar previous LPS-binding studies have only been conducted in mammalian cells (Triantafilou et al., 2000; Lalancette-Herbert et al., 2010).

Post-isolation, the influence of temperature on protoplasts is related to events that occur in the plasma membrane. Changes in the fluidity of the membrane, osmotic potential, protein denaturation and expansion-induced-lysis are some of the events that occur in plant cell membranes at low and high temperatures (Thomashaw, 1999; Salvucci et al., 2001). Any such changes would therefore have an effect on LPS*_E. coli_*-conjugate binding, and thus the optimal incubation temperature of treated protoplasts was investigated. All samples are reported relative to untreated protoplasts and protoplasts treated with non-labeled LPS (controls) which were set at 100%. De Filippis (1986) reported that elevated temperatures above 20°C produce a higher number of disrupted protoplasts. As in Supplementary Figure S3A, an incubation temperature of 37°C could have disrupted the protoplast cell membrane, thereby affecting the binding of Alexa-LPS*_E. coli_* to minimal levels as the fluorescence of these samples were not markedly higher than the untreated control and the LPS*_E. coli_*-treated protoplast sample. Lower temperatures also have adverse effects on the viability of protoplasts unless the cells have been acclimatized (Thomashaw, 1999), and may result from decreased enzyme activity. Incubation at 4°C improves protoplast half-life Zhang and Wong (2011), but upon incubation with a labeling agent, binding does not occur optimally and as seen in our results, Supplementary Figure S3B, which shows reduced binding of Alexa-LPS*_E. coli_* to the protoplasts. Unlike mammalian cells, plant protoplast binding was not found to be optimal at 37°C, neither at low temperatures. As such, RT was selected for subsequent binding site studies.

Ligand binding to protoplasts can also be affected by the incubation time(s) of the investigations. With shorter incubation periods at the concentrations used in this study, as selected due to the CAC and internalization considerations mentioned earlier, optimal binding cannot be expected as ligands may still be in solution searching for potential binding sites. In contrast with longer incubation times, ligands attach to binding sites and could possibly be endocytosed into protoplasts. Such considerations stimulated investigation of the optimal incubation time for conjugate-LPS*_E. coli_* binding.

**FIGURE 3:**
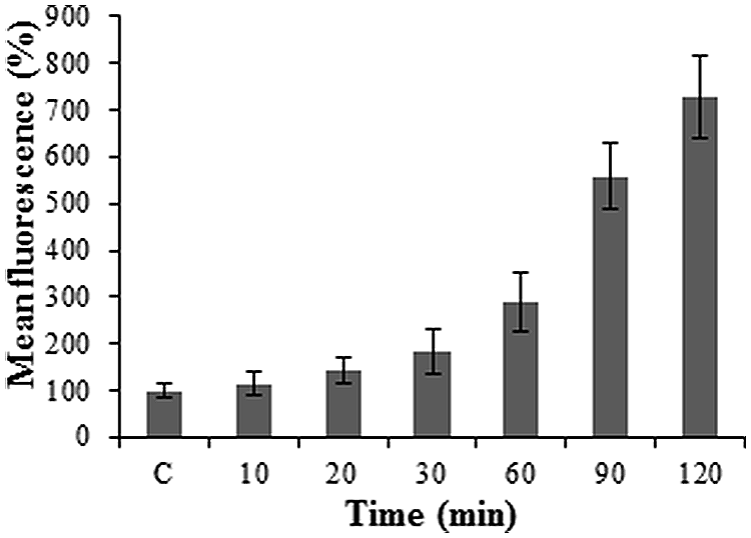
**Protoplast fluorescence changes over a 2 h incubation period following 0.4 μg/ml Alexa-LPS*_E. coli_* treatments at RT**. The control (C) represents unlabeled LPS*_E. coli_-*treated *Arabidopsis* mesophyll protoplasts set at 100%. Each data point represents the mean of three independent experiments and error bars the standard deviation thereof. The fluorescence levels remained constant after 2 h, followed by a decrease (data not shown).

**FIGURE 4:**
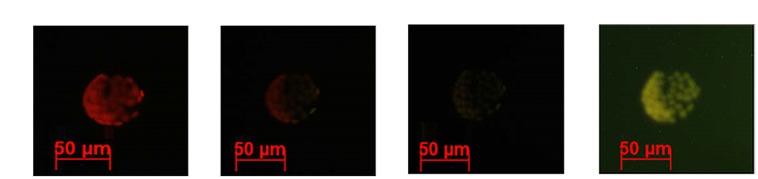
**Fluorescent micrographs indicating *Arabidopsis* protoplast fluorescence changes following treatment with 0.4 μg/ml Alexa-LPS*_E. coli_* over time, as viewed using filter set 09 (BP 450–490)**. Micrographs indicate Alexa-LPS*_E. coli_* binding to the protoplast surface at **(A)** 90 min, **(B)** 100 min, **(C)** 110 min, and **(D)** 120 min.

**Figure 3** illustrates a gradual increase in Alexa-LPS*_E. coli_* binding with time, with an ultimate sevenfold increase in fluorescence from the initial time (10 min) the cell membrane binds Alexa-LPS*_E. coli_* until the full 2 h incubation. This increase in fluorescence was attributed to an increased number of bound Alexa-LPS*_E. coli_* molecules. To support these results, fluorescent micrographs were also taken over the incubation time period. It was shown that the chosen concentration of Alexa-LPS*_E. coli_* optimally bound to the protoplast cell membranes between 110 and 120 min (**Figure 4**) which shows a completely green fluorescing protoplast cell membrane at 120 min. It was thus determined that an incubation time of 120 min was optimal for the cell membrane surface to become coated by fluorescing Alexa-LPS*_E. coli_*.

**FIGURE 5:**
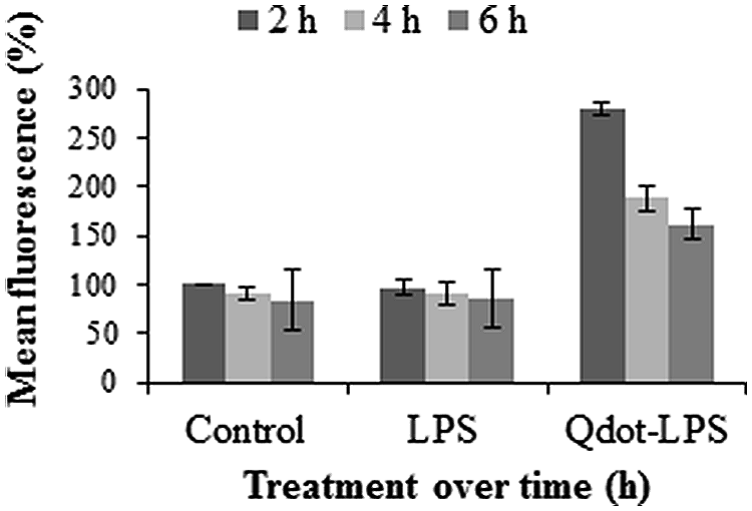
**Protoplast fluorescence changes over a 6 h incubation period following treatments with both unlabeled LPS (0.4 μg/ml LPS*_E. coli_*) and Qdot-LPS (0.46 μg/ml Qdot-LPS*_E. coli_*)**. Untreated *Arabidopsis* protoplasts were used as a control and set to a value of 100% (at 2 h) relative to which treated samples are reported. Three independent experiments were conducted and the error bars represent standard deviation.

According to **Figure 5**, the fluorescence of the controls decrease slightly but steadily with time. This can be ascribed to a gradual loss in protoplast viability over time and subsequent degradation/disintegration of the protoplast cells as seen in **Figure 1B**, which indicates a slight loss of viable protoplasts at 6 h. **Figure 5** furthermore shows protoplasts treated with Qdot-LPS*_E. coli_* to have a high fluorescence after 2 h of incubation with a steep decline at 4 h and progressively to 6 h. In this case, the control and non-labeled LPS treatments resulted in similar profiles and correlates well for these samples, while the Qdot-LPS*_E. coli_* samples showed the highest fluorescence as was expected. Qdots alone are highly stable fluorophores (Jaiswal and Simon, 2004; Müller et al., 2006; Betanzos et al., 2009) but the larger the shell structure (in this case due to conjugation to LPS), the lower the stability. Furthermore, fluorescence intensity is dependent on time (Sapsford et al., 2006). From these results we deduced that maximal fluorescence from conjugate-LPS*_E. coli_* binding occurs between 0 and 2 h, and thus a 2 h incubation period was selected for all subsequent experiments.

### Protoplast Binding Studies

During the optimization studies it was observed that LPS exhibited concentration-dependent aggregation (Aurell and Wistrom, 1998) that may mask epitopes of importance for binding at high concentrations. In addition, clumping of protoplasts at high concentrations due to bound LPS was also observed that negatively affected the flow cytometry detection (data not shown). Due to these experimental constraints, a classic ligand-receptor study could not be performed.

### Dose-Response Relationship of Labeled-LPS*_E. coli_* Binding to Protoplasts

**FIGURE 6:**
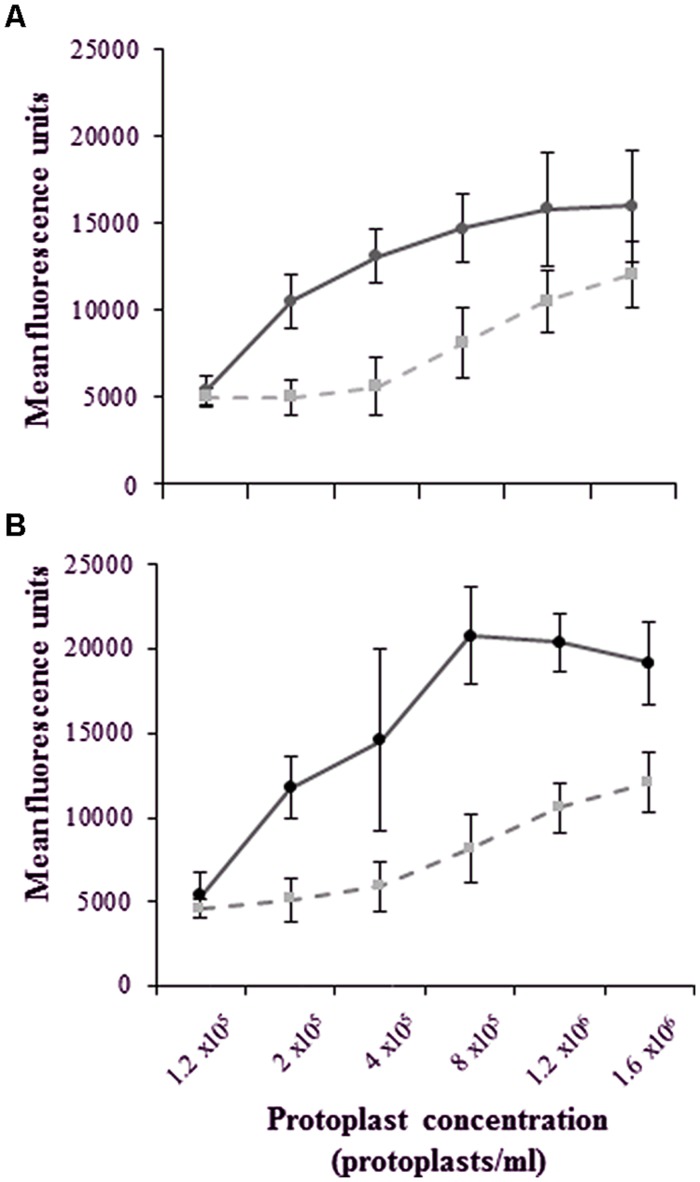
**Dose-dependent kinetics illustrating a plateau in the mean fluorescence even with an increase in the total number of binding sites on an increasing number of protoplasts following a 2 h treatment with **(A)** Alexa-LPS*_E. coli_* (measured at 500–560 nm) and **(B)** Qdot-LPS*_E. coli_* (measured at 590–630 nm)**. The unlabeled LPS*_E. coli_-*treated protoplasts as controls (dashed lines) are compared to treated protoplasts (solid lines), with the error bars representing the standard deviation of three independent experiments.

For these studies, an Alexa-LPS*_E. coli_* concentration of 0.40 μg/ml and Qdot-LPS*_E. coli_* of 0.46 μg/ml, respectively, was used throughout. **Figures 6A,B** show that with an increased number of protoplasts (1.2 × 10^5^ – 1.6 × 10^6^ protoplasts/ml) and thus available binding sites, there is also an increase in the number of bound, labeled-LPS*_E. coli_* molecules. This, however, reaches a plateau at the higher protoplast concentrations, thereby generating a hyperbolic pattern. In comparison, the unlabeled LPS*_E. coli_-*treated protoplasts (controls) indicate a steady increase in flow cytometry measurements due to chlorophyll autofluorescence.

Here, the plateau indicates the point where the selected, optimal conjugate-ligand concentration becomes rate-limiting, thus supporting an optimal protoplast working concentration of 2 x 10^5^/ml that falls within the exponential phase of dose-dependent measurements. Furthermore, this data indicates that both labeled ligands do bind to mesophyll protoplasts, regardless of whether the fluorescent conjugates were covalently or non-covalently (hydrophobically) linked. This was also confirmed when protoplasts were omitted and no fluorescence could be detected solely from the conjugates (data not shown). However, the higher fluorescence values of the Qdot-conjugate in comparison to that of autofluorescence within the exponential dose-dependent phase illustrates such labeling to be more sensitive than Alexa-labeling.

### Reversibility of the Labeled-LPS*_E. coli_* Binding in Protoplasts

Reversibility of binding is one of the criteria by which binding sites/receptors are defined (Smith et al., 1983). An excess of free, competitive ligand in the order of 100 times magnitude is required to interact with the binding sites of interest followed by addition of the labeled ligand at a set protoplast concentration. Accordingly, unlabeled LPS*_E. coli_* was added at different concentrations (in a pre-incubation step, Triantafilou et al., 2000), followed by addition of the respectively labeled LPS*_E. coli_-*conjugates at a protoplast concentration of 2 × 10^5^/ml in order to elucidate whether the ligand interacts reversibly with a binding site/receptor.

**FIGURE 7:**
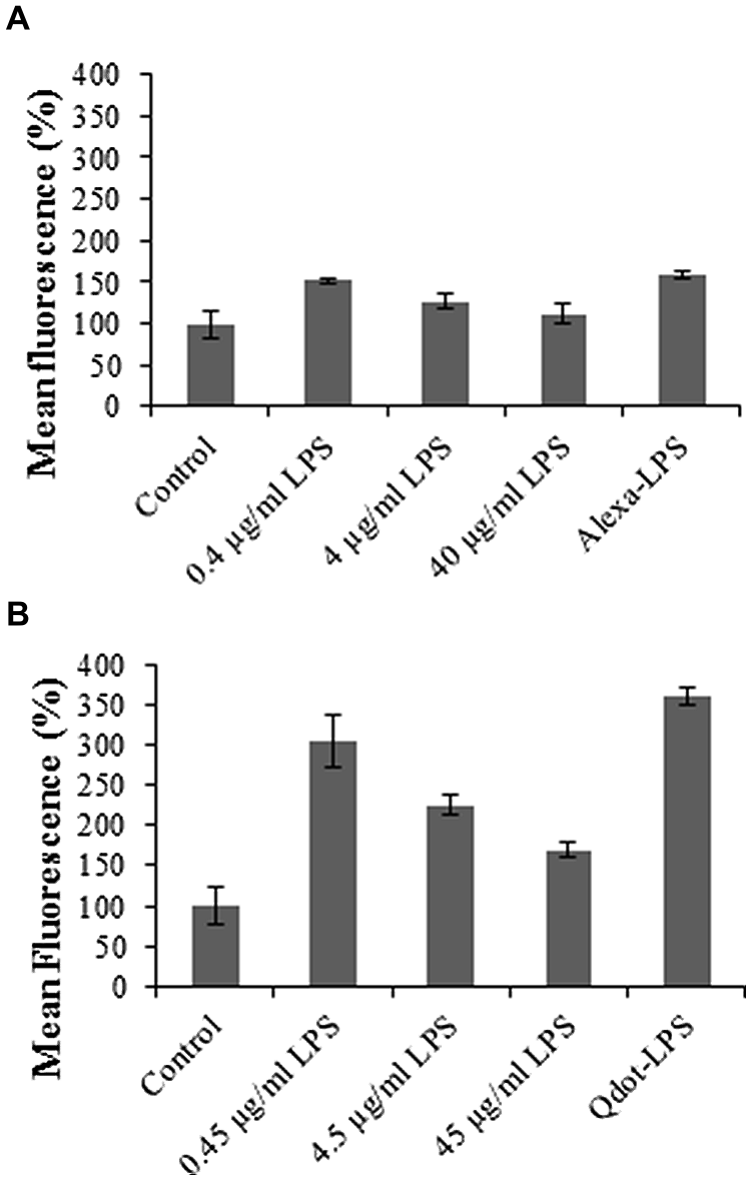
**Graphs illustrating binding reversibility of LPS-conjugates to mesophyll *Arabidopsis* binding sites**. Protoplasts were pre-incubated for 30 min with different concentrations of unlabeled LPS*_E. coli_*, as indicated, followed by treatment with **(A)** 0.4 μg/ml Alexa-LPS*_E. coli_* and **(B)** 0.46 μg/ml Qdot-LPS*_E. coli_*. Sole treatment (without unlabeled LPS pre-incubation) with labeled LPS-conjugates also served as positive controls. The negative controls represent unlabeled LPS*_E. coli_-*treated *Arabidopsis* mesophyll protoplasts set at 100%, with error bars representing standard deviation of three independent biological repeats.

The relative fluorescence of the conjugate (both Alexa- and Qdot- LPS*_E. coli_*) treatment alone is markedly higher in the case of the Qdot-labeled studies, but less pronounced in the Alexa-labeled studies. This may be ascribed to a more sensitive response from a more superior labeling strategy. The obtained results, however, showed an attenuation of fluorescence upon increase in the concentration of unlabeled LPS*_E. coli_* treatment. This illustrates that free LPS competes with labeled LPS*_E. coli_* (both Alexa- and Qdot-labeled binding), and thus the response obtained for the three competitive concentrations is representative of the total ligand binding. In this regard, Gross et al. (2005) also reported that the uptake of LPS could be outcompeted by the addition of an excess of unlabeled LPS in *N*. *tabacum* cells as was observed in **Figure 7**. Upon labeled LPS*_E. coli_* treatment, the conjugate binds to the few unbound/available binding sites and thus a low signal intensity is obtained at the higher concentrations of unlabeled LPS treatment. Triantafilou et al. (2000) similarly investigated specificity, with 100x unlabeled LPS pre-treatment inhibiting the binding of Alexa-LPS*_E. coli_*, thus proving specific binding to CHO cells. Scott et al. (2009) also showed that mouse hepatocytes are blocked due to pre-treatment with LPS. Although the studies were in mammalian cells, the same principle applies to plant cells. The response thus determined in **Figure 7** indicates reversible binding, with Qdot fluorophores proving to be a more sensitive labeling strategy.

Ultimately, these results with both labeled LPS-conjugates also pose the question as to whether unlabeled LPS pre-treatment leads to internalization of binding sites by ligand-dependent receptor-mediated endocytosis (RME), where LPS-binding sites prior to labeled LPS*_E. coli_* treatment are no longer available, and hence fewer binding sites. Alternatively, if not endocytosed, most binding sites are most likely occupied by the high concentrations of unlabeled LPS*_E. coli_*. Upon labeled LPS*_E. coli_* treatment, the conjugate binds to the few available (unbound) binding sites/receptors and a low signal intensity is obtained.

### Exo- and Endocytosis Inhibitors in Labeled LPS*_E. coli_* Binding Site Studies

Endocytosis occurs when molecules and/or receptors are translocated into the cell cytoplasm from the extracellular environment. Besides its role in signal attenuation by regulating the levels of ligand or available receptors, the process also exists in all cells so as to transport large polar molecules past the hydrophobic cell membrane and into the cell (Emans et al., 2002; Irani and Russinova, 2009). Both pinocytosis/fluid phase endocytosis (FPE) and/or RME are capable of receptor internalization (Aniento and Robinson, 2005). FPE is the uptake that occurs without clathrin-coated caveolae while RME requires clathrin-coated caveolae (Emans et al., 2002). Such endocytosis has been shown in the interaction of flg22 with FLS2, a LRR-RLK (Robatzek et al., 2006; Geldner and Robatzek, 2008; Beck et al., 2012a,b). Here, translocation of the complex from early endosomes to recycling endosomes for the FLS2 co-receptors, Brassinosteroid-Insensitive 1 (BRI1) and BRI1-associated-receptor kinase1 (BAK1), which are constitutive ligand receptors, occurs (Geldner and Robatzek, 2008; Frei dit Frey and Robatzek, 2009; Antolin-Llovera et al., 2012). Alternatively, endosomes marked for degradation in vacuoles by lytic enzymes commonly occurs in the case of ligand-induced RME of flg22-FLS2 (Geldner and Robatzek, 2008; Frei dit Frey and Robatzek, 2009; Irani and Russinova, 2009). Here, we report on Wortmannin and BFA as compounds capable of inhibiting endo- and exocytosis in plant cells, where the use of protoplasts is a feasible option to study the mode of endocytosis, albeit not, of LPS binding sites/receptors in plants. In this regard, BFA, is capable of inhibition of exocytosis (recycling) by targeting adenosine diphosphate ribosylation factor (ARF) guanine nucleotide exchange factor (GEF; Frei dit Frey and Robatzek, 2009), while Wortmannin, a potent endocytosis inhibitor, prevents the uptake of both receptors and ligands into the cell by specifically inhibiting phosphatidylinositol 3-kinase (PI3K; Shpetner et al., 1996; Wang et al., 2009). Endocytosis and signaling are, furthermore, said to be functionally interconnected as they share common molecular components on the plasma membrane (Geldner and Robatzek, 2008; Irani and Russinova, 2009; Beck et al., 2012a).

**FIGURE 8:**
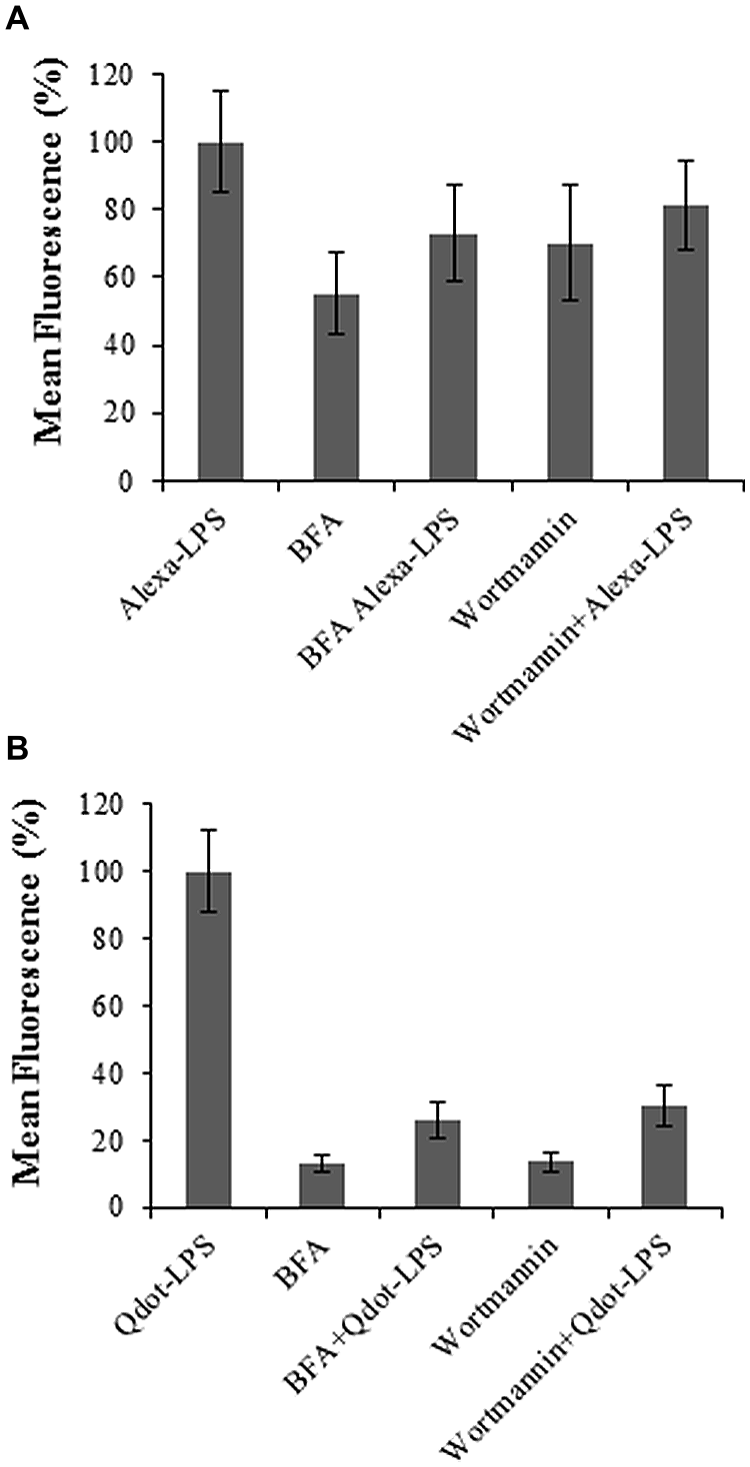
**The role of exo- (BFA) and endocytosis (Wortmannin) inhibitors in protoplasts treated with labeled LPS-conjugates**. Samples were co-treated simultaneously with either inhibitor and **(A)** 0.4 μg/ml Alexa-LPS*_E. coli_* and **(B)** 0.46 μg/ml Qdot-LPS*_E. coli_* respectively, followed by incubation at RT for 120 min. Sole treatment with each LPS-conjugate served a positive control and set to 100%, while BFA and Wortmannin sole treatments were included as negative controls. Error bars represent the standard deviation of three independent biological repeats.

It is hypothesized that initial LPS-conjugate binding takes place on the protoplast surface membrane and remains on surface binding sites/receptors upon Wortmannin treatment (**Figures 8A,B**) as a consequence of the inhibitor’s ability to prevent endocytosis. Thus only “initial binding sites” are measured as seen by the slight increased fluorescence of co-treated samples compared to the negative control, but much lower than the positive control. BFA, on the other hand, prevents exocytosis and thus inhibits recycling of such binding sites/receptors, leading to a decrease in fluorescence (compared to the positive controls) over time (**Figures 8A,B**), since again most likely only “initial binding sites” are measured.

Results obtained using these inhibitors exhibit characteristics of binding and perception of LPS*_E. coli_* as shown by reduced levels in fluorescence of the co-treated samples in comparison to those treated with only LPS conjugates. This suggests that when LPS binds to protoplasts, it may be internalized into the cell by endocytosis leading to transiently reduced levels of the binding site protein(s) on the surface. Gross et al. (2005) reported the specific recognition of LPS from *X. campestris* pv. *campestris* (LPS*_X.c.c_*) in *N. tabacum* cells. LPS was shown to be internalized into the cells when labeled with FITC in a temperature- and energy-dependent manner. Furthermore, endocytosis in plant cells was reported in the same study with the use of amantidine, an inhibitor of RME. Immunolocalization studies further proved co-localization of the LPS-elicitor with endosomal structures. In contrast, Zeidler et al. (2010) did not observe internalization during mobility studies of Alexa-labeled LPS*_S.__minnesota_* in *Arabidopsis* leaves.

When looking at the overall profile of **Figure 8** when compared to the work of Beck et al. (2012b), the BFA inhibition studies show a more sensitive response than the Wortmannin counterpart. This poses the question as to whether the recognition and subsequent endocytic route in this case may more resemble that of a non-activated ligand status (as is the case for the non-activated FLS2 receptor), i.e., a constitutive recycling. However, when comparing the profiles between **Figures 8A,B** specifically, it could be extrapolated that the Qdot-labeled studies present results which more resemble an activated ligand status in terms of endo- and exocytosis routes. This possibly highlights the difference and importance of the LPS-labeling strategy. Since the binding site/receptor and co-receptor complexes are not known in this case, the data and interpretation is currently speculative.

## Conclusion

Here, we demonstrate the use of Qdots, reported by Betanzos et al. (2009), as a preferential LPS-labeling strategy in comparison to previously used alternatives in binding studies. Triantafilou et al. (2000) appraised Alexa-labeling of LPS as a superior strategy in comparison to FITC, and so we compared Alexa to Qdots. The hydrophobic nanoparticles were successfully conjugated to LPS*_E. coli_*, and resulted in sensitive and bio-compatible Qdot-ligand complexes as previously reported (Sapsford et al., 2006; Yu et al., 2006a,b; Betanzos et al., 2009; Barr et al., 2010). Furthermore, another clear disadvantage of Alexa-labeling is the requirement of smooth LPS for covalent chemical modification which is not the case with Qdot-labeling. This illustrates the superiority of the latter for use in variant, including rough, LPS-binding site/receptor studies in plants.

Current models for MAMP perception predict the existence of PRRs (Zipfel, 2014). Although there is overlap in the cellular responses induced by LPS and other MAMPs that signal through surface receptors, a similar receptor/receptor complex has, however, not yet been reported for LPS. In this regard, Sanabria et al. (2012) proposed a role for S-domain RLKs in LPS perception and subsequent signal transduction events.

In general, the structural features of a ligand that determines the affinity for a receptor may be distinct from those which determine activity. Hence, the possibility exists that the carbohydrate and lipid moieties of LPS may be perceived by individual albeit interconnected or linked mechanisms (Madala et al., 2012). In this study, it was shown for the first time that specific binding sites for LPS do occur on *Arabidopsis* mesophyll protoplast surfaces using both Alexa-LPS*_E. coli_* and Qdot-LPS*_E. coli_*. Wortmannin investigations showed that LPS-binding most likely involves ligand-induced endocytotic process, while BFA studies furthermore hinted toward exocytosis-mediated recycling of the binding sites that may function in concert in the perception of LPS.

## Conflict of Interest Statement

The authors declare that the research was conducted in the absence of any commercial or financial relationships that could be construed as a potential conflict of interest.

## Author Contributions

LP and ID designed the research; LM performed the research; and LP, ID, and LM wrote the paper.
