# Supplementary material for: Comparative conventional- and quantum dot-labeling strategies for LPS binding site detection in Arabidopsis thaliana mesophyll protoplasts
Source: Front Plant Sci. 2015 May 12;6:335. doi: 10.3389/fpls.2015.00335 (PMC4428080; doi:10.3389/fpls.2015.00335)
Supplement: Supplementary file 1 [file Data_Sheet_1.DOCX]

***Supplementary Material***

**Comparative conventional- and quantum dot-labelling strategies for LPS binding site detection in *Arabidopsis thaliana* mesophyll protoplasts*.***

Londiwe S. Mgcina^1^, Ian A. Dubery^1^ and Lizelle A. Piater^1*^

^1^Department of Biochemistry, University of Johannesburg, Auckland Park, Johannesburg, South Africa, 2006

*** Correspondence:** Dr. LA Piater, University of Johannesburg, Department of Biochemistry, Cnr. Kingsway and University Road, Auckland Park 2006, Johannesburg, South Africa, [lpiater@uj.ac.za](mailto:lpiater@uj.ac.za)


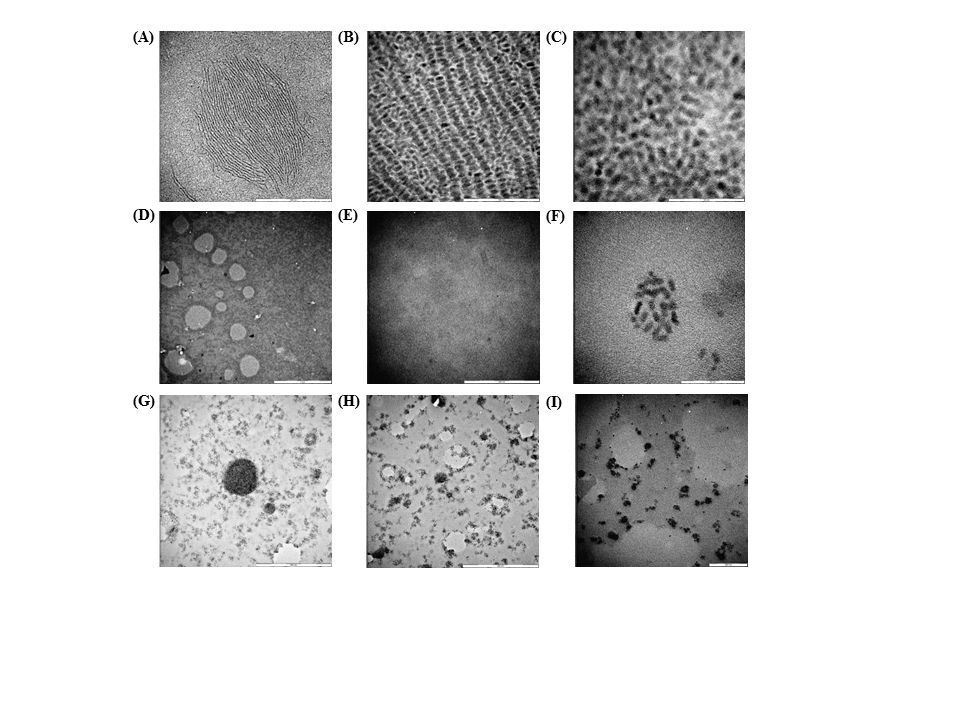


**Figure S1: Transmission Electron Micrograph (TEM) images** of **(A)** Qdots in chloroform (bar represents 500 nm), **(B)** Qdot in chloroform (bar represents 100 nm), **(C)** Qdot in chloroform (bar represents 50 nm), **(D)** 10 µg/ml LPS*_E. coli_* in dH_2_O (bar represents 2 µm) where micelles can be visualised due to the amphipathic nature of LPS*_E. coli_*, **(E)** 10 µg/ml LPS*_E. coli_* in dH_2_O (bar represents 200 nm) where a micelle was visualised at high magnification, **(F)** hydrophobic Qdot-LPS*_E. coli_* conjugates in water, showing the incorporation of hydrophobic Qdots in LPS*_E. coli_* micelles, deposited on a formvar-coated grid (bar represents 50 nm), **(G)** hydrophobic Qdot-LPS*_E. coli_* conjugates in water, showing the incorporation of hydrophobic Qdots in LPS micelles, deposited on a formvar-coated grid(bar represents 500 nm), **(H)** hydrophobic Qdot- LPS*_E. coli_* conjugates in water, showing the incorporation of hydrophobic Qdots in LPS micelles, deposited on a formvar-coated grid (bar represents 500 nm), and **(I)** hydrophobic Qdot-LPS*_E. coli_* conjugates in water, showing the incorporation of hydrophobic Qdots in LPS*_E. coli_* micelles, deposited on a formvar-coated grid (bar represents 200 nm).


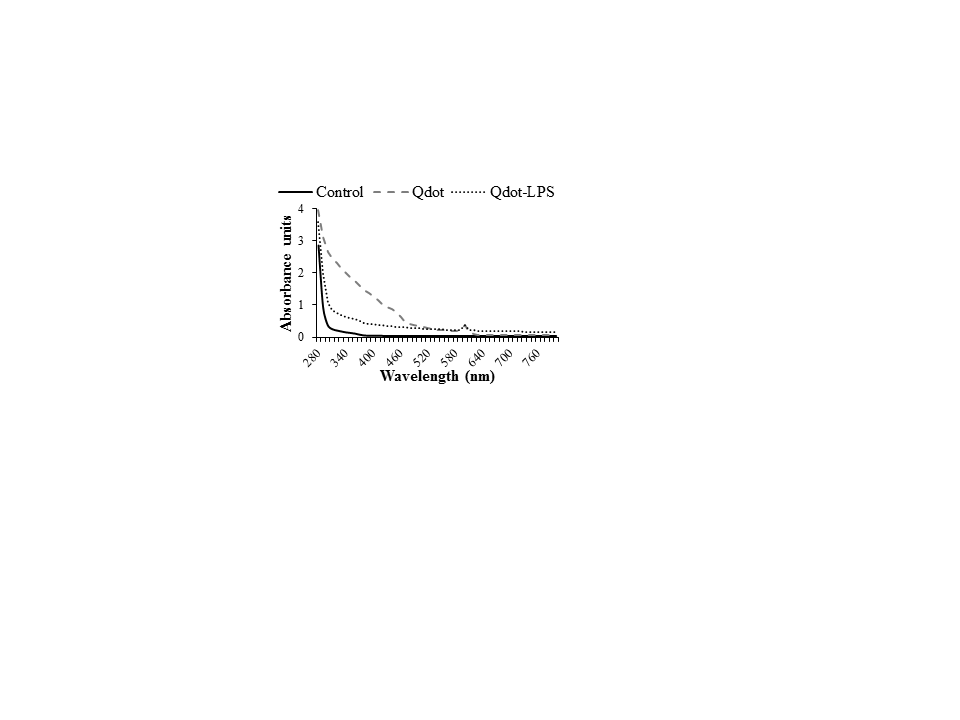


**Figure S2: UV/Vis spectra of Quantum dots (Qdot), Quantum dot-LPS*_E. coli_* (Qdot-LPS) and dH_2_O control.**


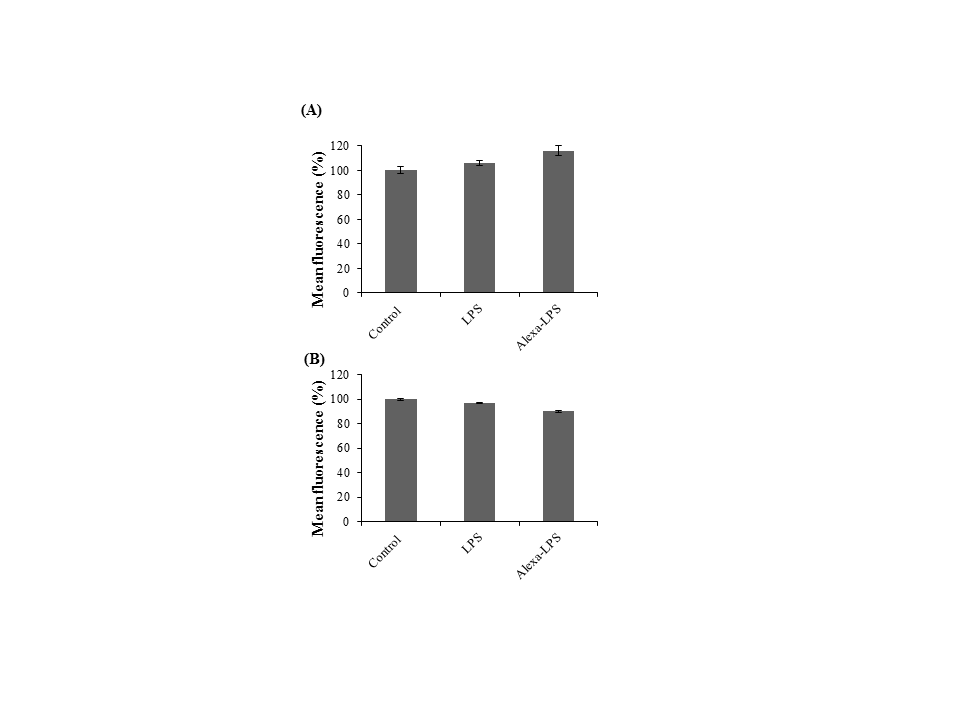


**Figure S3: The influence of temperature change on protoplasts** with incubation at **(A)** 37˚C and **(B)** 4˚C following a 90 min treatment of unlabeled leaf protoplasts (control), 10 µg/ml LPS*_E. coli_* treatment and protoplasts labelled with 0.4 µg/ml Alexa-LPS*_E. coli_*. The control represents untreated Arabidopsis mesophyll protoplasts set at 100%. Each data point represents the mean of three independent experiments and error bars the standard deviation thereof.
